# Supplementary material for: Foraging distance distributions reveal how honeybee waggle dance recruitment varies with landscape
Source: Commun Biol. 2024 Oct 11;7:1306. doi: 10.1038/s42003-024-06987-9 (PMC11470012; doi:10.1038/s42003-024-06987-9)
Supplement: Supplementary file 2 — Supplementary Information [file 42003_2024_6987_MOESM2_ESM.pdf]

Supplementary Information to

FORAGING DISTANCE DISTRIBUTIONS REVEAL HOW HONEYBEE WAGGLE DANCE RECRUITMENT VARIES WITH LANDSCAPE

JOSEPH PALMER, ASH E. SAMUELSON, RICHARD J. GILL, ELLOUISE LEADBEATER & VINCENT A. A. JANSEN

Contents

Supplementary Methods 1
Supplementary Table 1 6
Supplementary Table 2 7
Supplementary Figure 1 8
Supplementary Figure 2 9

Supplementary Methods

We will formulate a model to describe the distribution of distances reported on the dance floor. We first give a description of the scout and recruit dances, which we then combine in a full model for the distribution of dances of different lengths on the dance floor. As the full model tends to overfit we also derive a simpler model that has fewer parameters. We also and deal with the case where the data have a minimal observable value.

For the model we assume that there are n different resources and they are distributed according to a spatial Poisson point process. The Poisson point process places food sources randomly in the environment of the hive with intensity λ_i for resource i. The quality of that resource we will denote q_i. For convenience, we will order the resources q_i from small to large such that $q_j \geq q_i$ if $j > i$, and q_n is the resource with the highest quality, which we assume is always present in the environment, hence $\lambda_n > 0$, and has a resource quality exceeding unity, hence $q_n > 1$. Under this ordering, we define m, for which $1 \leq m \leq n$, as the lowest index for which $q_m > 1$ hence, $q_i < 1$ for all $i = 1, \ldots, m-1$.

The distribution of scout dances. As a scout travels a small distance $\mathrm{d}x$ its search will cover an area $c_s\,\mathrm{d}x$. The constant c_s depends on width of the area scanned, the detection rate and the degree to which a the forager's path covers new ground. If we incorporate these factors into the constant c_s the intensity of discovering a resource over a path of distance $\mathrm{d}x$ is $c_s\,\mathrm{d}x$. The probability density of x, the distance travelled after which the first source is discovered follows a distribution

$$\lambda_s e^{-\lambda_s x}$$

where $\lambda_s = c_s \sum_{i=1}^{n} \lambda_i$.

The profitability, which depends on quality and distance, of the source dictates the number of repeats of the dance. What is the distribution of dances on the dance floor? Let's assume that there is a function that translates the profitability into the expected number of runs of the waggle dance, $\phi(q_i, x)$, which is a function of quality an distance. We assume that if this function has a non-zero value it decreases with distance, and increases with quality, hence $\frac{\partial\phi(q,x)}{\partial q} > 0$ and $\frac{\partial\phi(q,x)}{\partial x} < 0$ if $\phi(q, x) > 0$. There is a minimum quality level of the resource that elicits dancing, and we have set this level to unity, such that if $q \leq 1$ then $\phi(q, x) = 0$ for $x \geq 0$. It follows that for each $q_i > 1$ there is a maximum distance $x_{i,\max} > 0$ for which bees do not dance, that is $\phi(q_i, x) = 0$ for $x \geq x_{i,\max}$.

The expected number of dances for a resource of type i located at distance x is $c_s\lambda_i\phi(q_i, x)e^{-\lambda_s x}$. The expected total number of scout waggle dances on the dance floor is given by

$$M_s = \sum_{i=m}^{n} \int_0^\infty \frac{c_s\lambda_i}{\lambda_s}\phi(q_i, x)\lambda_s e^{-\lambda_s x}\,\mathrm{d}x$$

and the distribution of scout dances is

$$\frac{\lambda_s e^{-\lambda_s x}\sum_{i=m}^{n}\frac{c_s\lambda_i}{\lambda_s}\phi(q_i, x)}{M_s}$$

If the bees go out, scout for resources and dance for these, and assuming there is no further processing of information, this would be the distribution one would expect.

The distribution of recruit dances. First, we consider a simple scenario and we assume that all resources are of equal quality q with $q > 1$. The scouts that report back on the dance floor cover the surroundings of the hive in their forays. How well they cover, depends on the number of scouts, how well their collective paths cover the area and chance of discovery of an item. Let the fraction of sources that can be discovered by the collection of scouts be c_r. We assume $0 \leq c_r \leq 1$.

The chance of discovering food source within a radius of x is $e^{-c_r\lambda_r\pi x^2}$, where $\lambda_r = \sum_{i=1}^{n}\lambda_i$ and therefore the chance of having the nearest food source that the recruits report is least a distance x away is $P(\underline{x} \geq x) = e^{-c_r\lambda_r\pi x^2}$. The distribution of the nearest food source that recruits report is

$$2c_r\lambda_r\pi x e^{-c_r\lambda_r\pi x^2}.$$

The distribution of recruit dances on the dance floor is:

$$\frac{\phi(q, x)2c_r\lambda_r\pi x e^{-\lambda_r\pi x^2}}{M_r}$$

where

$$M_r = \int_0^\infty \phi(q, x)2c_r\lambda_r\pi x e^{-c_r\lambda_r\pi x^2}\,\mathrm{d}x.$$

If the resources differ in quality, the same rationale can be applied. Consider two locations that differ in resource quality and distance, the hive can prefer the further location if it is sufficiently better. Likewise, it can prefer a lesser quality location if it is sufficiently nearer. Given that the hive has found a resource at distance x of quality q_i we

define a threshold distance $\xi_{ij}(x)$ at which a resource of type q_j has the same profitability. If $q_j \geq q_i$, and $0 \leq x \leq x_{i,\max}$ then $\xi_{ij}(x)$ satisfies

$$\begin{cases} \phi(q_i, x) = \phi(q_j, \xi_{ij}(x)) & \text{if } 0 \leq x < x_{i,\max} \\ \xi_{ij}(x_{i,\max}) = x_{j,\max} & \text{if } x = x_{i,\max} \end{cases}$$

If $q_j < q_i$, and $x < x_{i,\max}$ then ξ_{ij} satisfies

$$\begin{cases} \xi_{ij}(x) = 0 & \text{if } 0 \leq x \leq \xi_{ji}(0) \\ \phi(q_i, x) = \phi(q_j, \xi_{ij}(x))) & \text{if } \xi_{ji}(0) < x_{i,\max} \\ \xi_{ij}(x_{i,\max}) = x_{j,\max} & \text{if } x = x_{i,\max} \end{cases}$$

This relation defines the threshold distance $\xi_{ij}(x)$ as a function of x for $0 \leq x \leq x_{i,\max}$. It follows that $\xi_{ii}(x) = x$. Because $\phi(q_i, x)$ increases with q if $\phi(q_i, x) > 0$ it follows that $\phi(q_i, x) > \phi(q_j, x)$ if $q_i > q_j$. As a consequence $\xi_{ij} < x$ if $i > j$ and $\xi_{ij} > x$ and if $i < j$.

The probability to find the nearest resource i at distance x is given by the Rayleigh distribution $2\pi c_r \lambda_i x e^{-\pi c_r \lambda_i x^2}$. For $x \leq x_{i,\max}$ the chance of there not being a source if type j within a radius of $\xi_{ij}(x)$, which is $e^{-\pi c_r \lambda_j \xi_{ij}(x)^2}$. The probability that resource i at location x, $0 \leq x \leq x_{i,\max}$ is the most profitable is therefore

$$P(\text{most profitable resource } i \text{ is at distance } x) = 2\pi c_r \lambda_i x e^{-\pi \sum_{j=1}^{n} \lambda_j c_r \xi_{ij}(x)^2}$$

and 0 if $x > x_{i,\max}$ and the probability of finding the most profitable location at distance x is

$$P(\text{most profitable resource is at distance } x) = \sum_{\forall i : x_{i,\max} > x} 2\pi c_r \lambda_i x e^{-\pi \sum_{j=1}^{n} c_r \lambda_j \xi_{ij}(x)^2}.$$

For $x > x_{n,\max}$ the most profitable resource cannot be determined, as all have zero profitability.

The probability of encountering a recruit reporting a distance x on the dance floor is

$$M_r^{-1} \sum_{i=m}^{n} \phi(q_i, x) \lambda_i 2\pi c_r x e^{-\pi \sum_{j=1}^{n} c_r \lambda_j \xi_{ij}(x)^2}$$

where M_r is the normalisation factor

$$M_r = \int_0^\infty \sum_{i=m}^{n} \phi(q_i, x) \lambda_i 2\pi c_r x e^{-\pi \sum_{j=1}^{n} c_r \lambda_j \xi_{ij}(x)^2} \, \mathrm{d}x.$$

The full model. The combined distribution of distances of scout and recruit dances on the dance floor is given by

$$P(\underline{x} = x) = p \frac{\lambda_s e^{-\lambda_s x} \sum_{i=1}^{n} \frac{c_s \lambda_i}{\lambda_s} \phi(q_i, x)}{M_s} + (1-p) \frac{\sum_{i=1}^{n} \phi(q_i, x) 2\pi c_r \lambda_i x e^{-\pi \sum_{j=1}^{n} c_r \lambda_j \xi_{ij}(x)^2}}{M_r}.$$

This is a generic model for the distribution of distances reported on the dance floor.

The simplified model. The full model can be used to calculate the likelihood for a given data set, which can be used to estimate parameters. The full model has $2n+3+k$ parameters: $\lambda_1 \dots \lambda_n$, $q_1 \dots q_n$, c_s, c_r and p plus k parameters needed to specify the function ϕ. Even though it is possible to reduce the number of parameters through a scaling, e.g. by defining $\lambda_i^r = c_r \lambda_i$ and $c = \frac{c_s}{c_r}$, but even if the number of resources is low, it turned out to be difficult to estimate the parameters even if with a fair sized data sets. To assist in the estimation of the key parameters we therefore formulated a simplified model.

We can simplify the model by assuming that the dependence of the number of dances depends less on the distance and more on quality. We assume that that dependence of the profitability assessment only weakly depends on distance and that there is quality differences between resources of a decent size, so that there are a large threshold distances at which resources have the same profitability. If that is the case then, if a resource is discovered at a small distance from the hive, resources if less quality than this one, are not worth dancing for, whereas there is a large distance over which superior resources can be detected that will have higher profitability. In mathematical terms, under this assumption, for small x, $\xi_{ij}(x) = 0$ if $j < i$, $\xi_{jj}(x) = x$ and $\xi_{ij}(x)$ is large if $j > i$. We thus have $\sum_{j=1}^n \lambda_j \xi_{ij}(x) = \sum_{j=i}^n \lambda_j \xi_{ij}(x)$ for $i < n$ and $\sum_{j=1}^n \lambda_j \xi_{nj}(x) = \lambda_n x$. Moreover, as the $\xi_{ij}s$ will be large, $\sum_{j=i}^n \lambda_j \xi_{ij}(x) \gg \lambda_n x$. We can approximate

$$\sum_{i=1}^{n} \phi(q_i, x) 2\pi c_r \lambda_i x e^{-\pi \sum_{j=1}^n c_r \lambda_j \xi_{ij}(x)^2} \approx \phi(q_n, x) 2\pi c_r \lambda_n x e^{-\pi c_r \lambda_n x^2}$$

The recruits on the dance floor are most likely to report short distances. By expanding the function $\phi(q_n, x)$ around $x = 0$ we get $\phi(q_n, x) = \left[\phi(q_n, 0) + x \left.\frac{\partial \phi(q_n,x)}{\partial x}\right|_{x=0} + O(x^2)\right]_+$ we can approximate the recruit distribution as:

$$M_r^{-1} \sum_{i=m}^{n} \phi(q_i, x) \lambda_i 2\pi c_r x e^{-\pi \sum_{j=1}^n c_r \lambda_j \xi_{ij}(x)^2} \approx \frac{[1 - a_r x]_+ 2\pi a_r^2 b_r x e^{-\pi b_r (a_r x)^2}}{1 - \frac{\operatorname{erf}\left(\sqrt{\pi b_r}\right)}{2\sqrt{b_r}}}$$

with $a_r = -\phi(q_n, 0)^{-1} \left.\frac{\partial \phi(q_n,x)}{\partial x}\right|_{x=0}$ and $b_r = c_r \gamma_n a_r^{-2}$ and where we used

$$\phi(q_n, 0) \int_0^{a_r^{-1}} (1 - a_r x) 2\pi a_r^2 b_r x e^{-\pi b_r (a_r x)^2} \, \mathrm{d}x = \phi(q_n, 0) \left(1 - \frac{\operatorname{erf}\left(\sqrt{\pi b_r}\right)}{2\sqrt{b_r}}\right)$$

and cancelled the term $\phi(q_n, 0)$.

The scouts on the dance floor are more likely to report large distances. For distances around the largest distance for which scouts dance, $x_{n,\max}$, the $\phi(q_i, x) = 0$ for all $i < n$. We therefore expand $\phi(q_n, x)$ around $x_{n,\max}$ to get $\phi(q_n, x) = \left[(x - x_{n,\max}) \left.\frac{\phi(q_n,x)}{\partial x}\right|_{x=x_{n,\max}} + O(x^2)\right]_+$. With this, we can approximate the scout distribution as:

$$\frac{\lambda_s e^{-\lambda_s x} \sum_{i=m}^n \frac{c_s \lambda_i}{\lambda_s} \phi(q_i, x)}{M_s} \approx \frac{a_s b_s e^{-b_s a_s x} [1 - a_s x]_+}{1 - b_s^{-1} (1 - e^{-b_s})}$$

with $a_s = x_{n,\max}^{-1}$ and $b_s = \gamma_s a_s^{-1}$ and where we used

$$\frac{c_s\lambda_i}{\lambda_s} \left.\frac{\phi(q_n,x)}{\partial x}\right|_{x=x_{n,\max}} \int_0^{a_s^{-1}} a_s b_s e^{-b_s a_s x}(1-a_s x)\,\mathrm{d}x = \frac{c_s\lambda_i}{\lambda_s} \left.\frac{\phi(q_n,x)}{\partial x}\right|_{x=x_{n,\max}} \left(1 - b_s^{-1}\left(1-e^{-b_s}\right)\right)$$

and cancelled the term $\frac{c_s\lambda_i}{\lambda_s} \left.\frac{\phi(q_n,x)}{\partial x}\right|_{x=x_{n,\max}}$.

The distribution then simplifies to

$$P(\underline{x}=x) = p\frac{\left[1-a_s x\right]_+ a_s b_s e^{-b_s a_s x}}{1-b_s^{-1}\left(1-e^{-b_s}\right)} + (1-p)\frac{\left[1-a_r x\right]_+ 2\pi a_r^2 b_r x e^{-\pi b_r (a_r x)^2}}{1-\frac{\operatorname{erf}\left(\sqrt{\pi b_r}\right)}{2\sqrt{b_r}}}.$$

The simplified model has 5 parameters, and is therefore simpler than the full model for $n \geq 2$, which has $2+2n+k$ parameters.

The simplified distribution with a minimum value. Let m be the lowest distance that is recorded, for instance because there is a minimum duration of the waggle dance. We assume that $m < \xi_{ji}(0)$ for all $j < i$. For the simplified recruit distribution we now expanding the function $\phi(q_n,x)$ around $x = m$ to get $\phi(q_n,x) = \left[\phi(q_n,m) + (x-m)\left.\frac{\partial\phi(q_n,x)}{\partial x}\right|_{x=m} + O(x^2)\right]_+$. The simplified recruit distribution becomes:

$$\frac{\left[1-a_r x\right]_+ 2\pi a_r^2 b_r x e^{-\pi b_r (a_r x)^2}}{\left(1-a_r m\right)e^{-\pi b_r (a_r m)^2} + \frac{\operatorname{erf}\left(a_r\sqrt{\pi b_r}m\right)-\operatorname{erf}\left(\sqrt{\pi b_r}\right)}{2\sqrt{b_r}}}$$

with $a_r = m - \phi(q_n,m)^{-1}\left.\frac{\partial\phi(q_n,x)}{\partial x}\right|_{x=m}$ and $b_r = c_r\gamma_n a_r^{-2}$.

The scout distribution is only affected through the normalisation factor and becomes:

$$a_s\frac{b_s e^{-b_s a_s (x-m)}\left[1-a_s x\right]_+}{\left(1-a_s m\right) - b_s^{-1}\left(1-e^{-b_s(1-a_s m)}\right)}$$

The distribution, for $x \geq m$ then becomes

$$\begin{aligned} P(\underline{x}=x) &= p a_s\frac{b_s e^{-b_s a_s (x-m)}\left[1-a_s x\right]_+}{\left(1-a_s m\right) - b_s^{-1}\left(1-e^{-b_s(1-a_s m)}\right)} \\ &\quad +(1-p)\frac{\left[1-a_r x\right]_+ 2\pi a_r^2 b_r x e^{-\pi b_r (a_r x)^2}}{\left(1-a_r m\right)e^{-\pi b_r (a_r m)^2} + \frac{\operatorname{erf}\left(a_r\sqrt{\pi b_r}m\right)-\operatorname{erf}\left(\sqrt{\pi b_r}\right)}{2\sqrt{b_r}}}. \end{aligned}$$

The same function describes the distribution of the dance durations, where dances have a minimum duration. This assumes a linear relation between duration and distance.

Supplementary Table 1

Fitting of the waggle dance model

	site	type	n_data	model	log l'hood	AIC	ΔAIC	rAIC	wAIC	p	b_s	b_r	a_s	a_r	k	ks_stat	ks_Pval
1	BEL	agri-rural	102	collective	-181.41	372.83	1.83	0.40	0.29	0.65	7.04	0.81	0.05	0.17	5	0.10	0.71
2	BEL	agri-rural	102	individual	-183.50	371.00	0.00	1.00	0.71	1.00	4.50		0.07		2	0.09	0.79
3	BFI	agri-rural	229	collective	-190.50	391.00	0.00	1.00	1.00	0.04	0.00	2.82	0.16	0.25	5	0.09	0.33
4	BFI	agri-rural	229	individual	-211.30	426.59	35.60	0.00	0.00	1.00	9.47		0.10		2	0.18	0.00
5	BLO	urban	170	collective	-221.03	452.05	0.00	1.00	1.00	0.21	1.79	0.57	0.07	0.36	5	0.07	0.76
6	BLO	urban	170	individual	-237.39	478.78	26.73	0.00	0.00	1.00	5505		0.00		2	0.11	0.24
7	BUR	urban	164	collective	-118.30	246.61	0.00	1.00	1.00	0.11	0.00	500.00	0.15	0.03	5	0.07	0.82
8	BUR	urban	164	individual	-143.72	291.44	44.83	0.00	0.00	1.00	10.00		0.10		2	0.18	0.01
9	CAD	urban	73	collective	-48.28	106.56	2.65	0.27	0.21	0.17	0.00	0.43	1.46	0.39	5	0.08	0.95
10	CAD	urban	73	individual	-49.95	103.91	0.00	1.00	0.79	1.00	0.00		0.39		2	0.11	0.74
11	GIL	urban	192	collective	-74.54	159.08	0.00	1.00	1.00	0.26	0.00	0.00	0.33	0.75	5	0.14	0.06
12	GIL	urban	192	individual	-102.31	208.62	49.53	0.00	0.00	1.00	2.04		0.35		2	0.20	0.00
13	HER	agri-rural	89	collective	-136.97	283.94	2.08	0.35	0.26	0.28	0.00	0.16	0.38	0.18	5	0.08	0.95
14	HER	agri-rural	89	individual	-138.93	281.86	0.00	1.00	0.74	1.00	0.00		0.18		2	0.11	0.59
15	HHS	urban	90	collective	-61.35	132.70	0.00	1.00	1.00	0.10	0.00	0.00	0.14	0.50	5	0.13	0.36
16	HHS	urban	90	individual	-83.75	171.50	38.80	0.00	0.00	1.00	7.10		0.13		2	0.24	0.01
17	HOR	urban	150	collective	-40.50	91.00	0.00	1.00	1.00	0.06	0.00	0.54	0.22	0.63	5	0.05	0.97
18	HOR	urban	150	individual	-56.65	117.30	26.30	0.00	0.00	1.00	7.77		0.20		2	0.17	0.02
19	MAK	urban	99	collective	-84.52	179.04	0.00	1.00	1.00	0.21	2.32	0.02	0.12	0.48	5	0.09	0.79
20	MAK	urban	99	individual	-98.02	200.05	21.01	0.00	0.00	1.00	9.17		0.10		2	0.19	0.03
21	MEL	agri-rural	123	collective	-115.32	240.63	0.00	1.00	1.00	0.08	0.00	0.00	0.21	0.34	5	0.16	0.07
22	MEL	agri-rural	123	individual	-135.73	275.46	34.83	0.00	0.00	1.00	0.00		0.25		2	0.24	0.00
23	MPA	urban	150	collective	-181.87	373.75	0.00	1.00	0.96	0.57	0.58	0.00	0.19	0.42	5	0.07	0.87
24	MPA	urban	150	individual	-188.04	380.08	6.33	0.04	0.04	1.00	1.47		0.19		2	0.10	0.43
25	ROT	agri-rural	194	collective	-138.13	286.26	0.00	1.00	1.00	0.31	0.00	0.00	0.31	0.48	5	0.15	0.02
26	ROT	agri-rural	194	individual	-153.36	310.71	24.45	0.00	0.00	1.00	0.00		0.35		2	0.20	0.00
27	SAU	agri-rural	132	collective	-108.18	226.37	0.00	1.00	0.99	0.31	0.00	9.98	0.26	0.16	5	0.06	0.96
28	SAU	agri-rural	132	individual	-115.50	235.01	8.64	0.01	0.01	1.00	1.49		0.27		2	0.13	0.21
29	SOM	agri-rural	113	collective	-70.54	151.07	0.00	1.00	0.96	0.00	10.00	10.00	1.50	0.15	5	0.11	0.50
30	SOM	agri-rural	113	individual	-76.67	157.34	6.27	0.04	0.04	1.00	0.00		0.38		2	0.16	0.10
31	SRA	agri-rural	154	collective	-123.37	256.74	0.00	1.00	1.00	0.24	0.00	0.21	0.21	0.49	5	0.05	0.98
32	SRA	agri-rural	154	individual	-138.11	280.23	23.49	0.00	0.00	1.00	3.00		0.21		2	0.14	0.07
33	STU	agri-rural	110	collective	-155.70	321.40	4.32	0.12	0.10	0.35	0.00	1.02	0.52	0.16	5	0.05	1.00
34	STU	agri-rural	110	individual	-156.54	317.08	0.00	1.00	0.90	1.00	1.35		0.16		2	0.05	1.00
35	SWP	urban	90	collective	-40.42	90.85	0.00	1.00	0.95	0.00	0.25	1.27	0.00	0.38	5	0.10	0.71
36	SWP	urban	90	individual	-46.44	96.88	6.03	0.05	0.05	1.00	0.00		0.42		2	0.14	0.31
37	YAL	agri-rural	182	collective	-205.76	421.52	0.00	1.00	1.00	0.08	0.00	0.30	0.18	0.27	5	0.04	0.99
38	YAL	agri-rural	182	individual	-222.43	448.87	27.35	0.00	0.00	1.00	0.22		0.21		2	0.10	0.34
39	ZSL	urban	221	collective	-110.01	230.02	0.00	1.00	1.00	0.24	0.00	0.79	0.39	0.50	5	0.04	1.00
40	ZSL	urban	221	individual	-119.95	243.90	13.88	0.00	0.00	1.00	0.39		0.42		2	0.10	0.25
41		urban	1399	collective	-1192.04	2394.09	0.00	1.00	1.00	0.11	5.12	10.00	0.07	0.16	5	0.04	0.14
42		urban	1399	individual	-1408.14	2820.27	426.19	0.00	0.00	1.00	26.31		0.04		2	0.17	0.00
43		agri-rural	1428	collective	-1674.34	3358.67	0.00	1.00	1.00	0.18	4.28	7.52	0.08	0.13	5	0.03	0.44
44		agri-rural	1428	individual	-1819.41	3642.81	284.14	0.00	0.00	1.00	7.44		0.09		2	0.15	0.00

Results for the fitting of the waggle dance model to individual sites are above the line, below the line are the results of the pooled data from urban and agri-rural sites. The number of data points for each fit is given by n_data, k indicates the number of parameters in the model. The value of the KS statistic is in the column ks_stat and the P-value under ks_Pval.

Supplementary Table 2

Area cover in land-use types

Environment	Land-use	% coverage
Agri-rural	Arable	28.30
Agri-rural	Pasture	23.30
Agri-rural	Woodland	21.10
Agri-rural	Built Up Area	15.00
Agri-rural	Fruit	3.10
Agri-rural	Oilseed Rape	2.90
Agri-rural	Non Agricultural Unmanaged Green Space	2.80
Agri-rural	Non Agricultural Managed Green Space	1.80
Agri-rural	Other Agricultural	1.50
Agri-rural	Water	0.20
Urban	Sparse Residential	34.80
Urban	Continuous Central	24.30
Urban	Dense Residential	21.80
Urban	Parks Allotments Cemeteries	7.90
Urban	Woodland	4.20
Urban	Water	3.40
Urban	Amenity Grassland	2.60
Urban	Railway	1.00

Percentage area covered for each land-use type in the agri-rural and urban environments in the sites studied.

Supplementary Figure 1

Cumulative frequency distributions

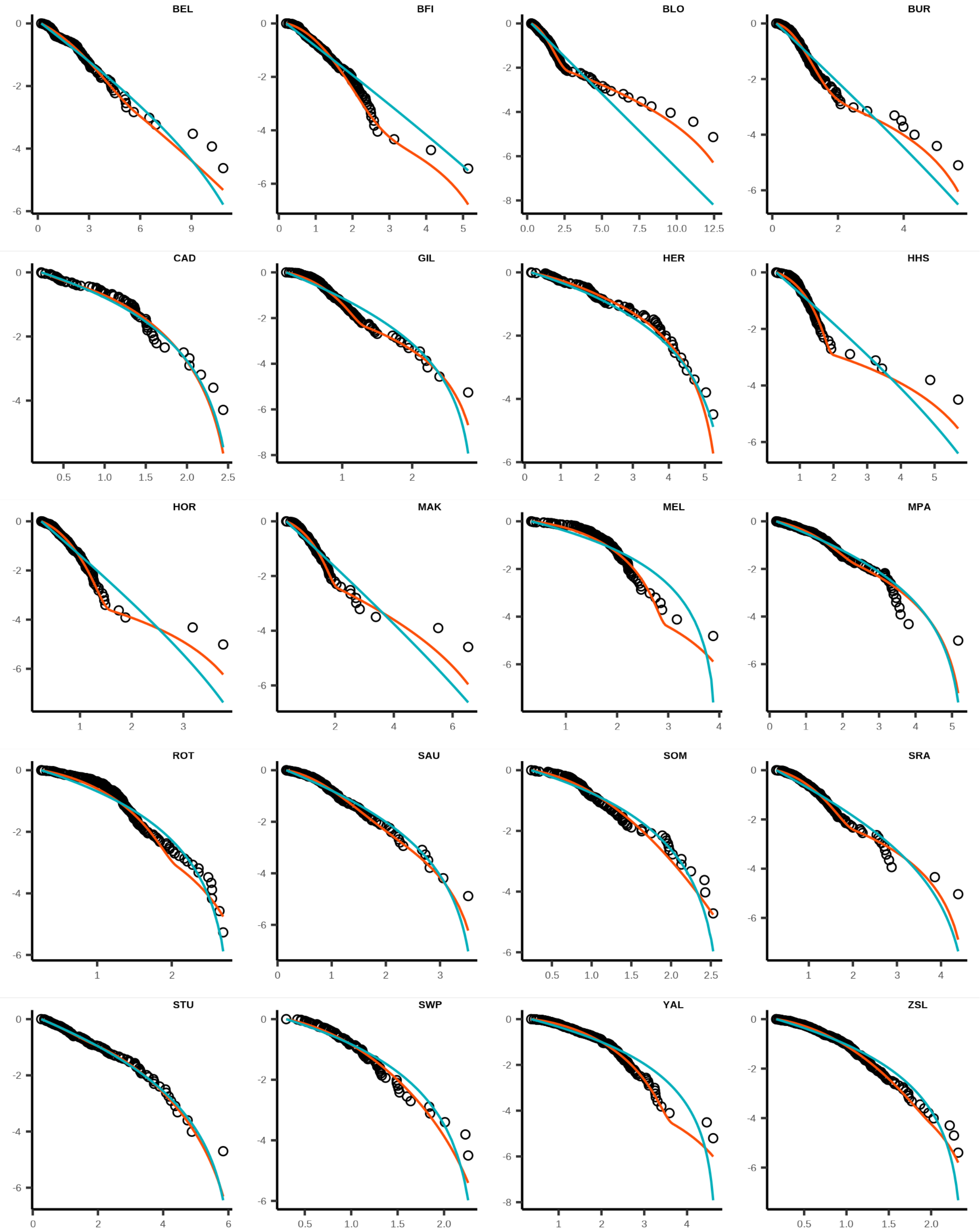

Cumulative frequency distribution of waggle dance durations and fits of the collective (red line) and individual (blue line) models for all 20 sites

Supplementary Figure 2

Jack-knifed Partial Least Squares Analysis.

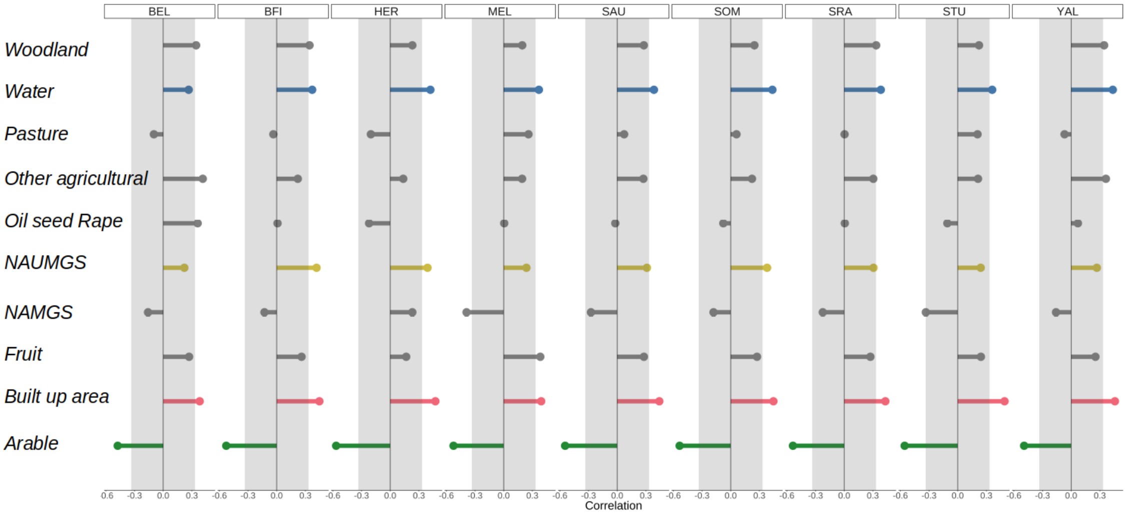

Loadings of PLS calculated with one site removed for the agri-rural sites. Each columns shows the loadings of the first principal component with a site (named at top of column) removed from the analysis, showing the individual points making up the overall box plot loadings in Fig 5c.
