# Supplementary material for: Foraging distance distributions reveal how honeybee waggle dance recruitment varies with landscape
Source: Commun Biol. 2024 Oct 11;7:1306. doi: 10.1038/s42003-024-06987-9 (PMC11470012; doi:10.1038/s42003-024-06987-9)
Supplement: Supplementary file 3 — Reporting Summary [file 42003_2024_6987_MOESM3_ESM.pdf]

nature portfolio

Corresponding author(s): Vincent Jansen

Last updated by author(s): Sep 14, 2024

Reporting Summary

Nature Portfolio wishes to improve the reproducibility of the work that we publish. This form provides structure for consistency and transparency in reporting. For further information on Nature Portfolio policies, see our Editorial Policies and the Editorial Policy Checklist.

Statistics

For all statistical analyses, confirm that the following items are present in the figure legend, table legend, main text, or Methods section.

n/a	Confirmed	
☐	☒	The exact sample size (*n*) for each experimental group/condition, given as a discrete number and unit of measurement
☐	☒	A statement on whether measurements were taken from distinct samples or whether the same sample was measured repeatedly
☐	☒	The statistical test(s) used AND whether they are one- or two-sided<br>*Only common tests should be described solely by name; describe more complex techniques in the Methods section.*
☐	☒	A description of all covariates tested
☐	☒	A description of any assumptions or corrections, such as tests of normality and adjustment for multiple comparisons
☐	☒	A full description of the statistical parameters including central tendency (e.g. means) or other basic estimates (e.g. regression coefficient) AND variation (e.g. standard deviation) or associated estimates of uncertainty (e.g. confidence intervals)
☐	☒	For null hypothesis testing, the test statistic (e.g. *F*, *t*, *r*) with confidence intervals, effect sizes, degrees of freedom and *P* value noted<br>*Give P values as exact values whenever suitable.*
☐	☒	For Bayesian analysis, information on the choice of priors and Markov chain Monte Carlo settings
☐	☒	For hierarchical and complex designs, identification of the appropriate level for tests and full reporting of outcomes
☒	☐	Estimates of effect sizes (e.g. Cohen's *d*, Pearson's *r*), indicating how they were calculated

Our web collection on statistics for biologists contains articles on many of the points above.

Software and code

Policy information about availability of computer code

Data collection	Waggle dance data was recorded by training a camcorder onto the dance floor. No special software was used for data collection. Details of the data collection methods can be found in Material and Methods and Samuelson et al. (2022) https//doi.org/10.1111/1365-2664.14011.
Data analysis	All analysis and code can be found on Zenodo https://doi.org/10.5281/zenodo.7025590 (2024) and on github: https://github.com/joseph-palmer/wagglefit

For manuscripts utilizing custom algorithms or software that are central to the research but not yet described in published literature, software must be made available to editors and reviewers. We strongly encourage code deposition in a community repository (e.g. GitHub). See the Nature Portfolio guidelines for submitting code & software for further information.

Data

Policy information about availability of data

All manuscripts must include a data availability statement. This statement should provide the following information, where applicable:

- Accession codes, unique identifiers, or web links for publicly available datasets
- A description of any restrictions on data availability
- For clinical datasets or third party data, please ensure that the statement adheres to our policy

Data availability statement included in manuscript

Research involving human participants, their data, or biological material

Policy information about studies with human participants or human data. See also policy information about sex, gender (identity/presentation), and sexual orientation and race, ethnicity and racism.

Reporting on sex and gender	NA
Reporting on race, ethnicity, or other socially relevant groupings	NA
Population characteristics	NA
Recruitment	NA
Ethics oversight	NA

Note that full information on the approval of the study protocol must also be provided in the manuscript.

Field-specific reporting

Please select the one below that is the best fit for your research. If you are not sure, read the appropriate sections before making your selection.

☐ Life sciences ☐ Behavioural & social sciences ☒ Ecological, evolutionary & environmental sciences

For a reference copy of the document with all sections, see nature.com/documents/nr-reporting-summary-flat.pdf

Ecological, evolutionary & environmental sciences study design

All studies must disclose on these points even when the disclosure is negative.

Study description	The study details a mathematical model to describe collective foraging by honeybees using the waggle dance and explores the model's fit to a published waggel dance data set. We estimate waggle dance use for hives in different locations and then explore the factors druiving dance use
Research sample	Waggle dance observations on honeybee hives were recorded in agri-rural and urban sites across South East England, as reported in Samuelson et al. (2022) https//doi.org/10.1111/1365-2664.14011. We chose this data set as it is a large sample of dances across multiple sites and so allowed us to fit our model to explore the effect of land use type on waggle dance use
Sampling strategy	Data sampling was carried out as described in Samuelson et al. (2022) https//doi.org/10.1111/1365-2664.14011
Data collection	The data was collected by A.E. Samuelson who recorded the dance floors and manually decoded the dances. The data used in this study are the waggle dance decodings of observations made in 2017, and were provided by Samuelson and Leadbeater. The full methodology of the data collection can be found in Samuelson et al. (2022) https//doi.org/10.1111/1365-2664.14011. Summary data were published on the Dryad Digital Repository https://doi.org/10.5061/dryad.c2fqz618f (Samuelson et al. 2021), and the full dataset can be found on Zenodo https://doi.org/10.5281/zenodo.7025590.
Timing and spatial scale	Dances from each site were videoed once every 2 weeks for 24 weeks between April and September 2017 (two sites visited each day between 8:00-12:00 and 12:00-17:00, respectively.) The full methodology of how the data was collected can be found in Samuelson et al. (2022) https//doi.org/10.1111/1365-2664.14011
Data exclusions	All of the data used collected between 24 weeks between April and September 2017 were used in this study. For some sites the original study collected data in 2016. To keep the reporting period consistent these data were not used in this paper. See Samuelson et al. (2022) https//doi.org/10.1111/1365-2664.14011 for further details.
Reproducibility	All of the code used to generate the results in this paper and the waggle dance data used in this study is provided through Zenodo (https://doi.org/10.5281/zenodo.7025590).
Randomization	NA
Blinding	Not relevant as we used data that was already collected

Did the study involve field work? ☐ Yes ☒ No

Reporting for specific materials, systems and methods

We require information from authors about some types of materials, experimental systems and methods used in many studies. Here, indicate whether each material, system or method listed is relevant to your study. If you are not sure if a list item applies to your research, read the appropriate section before selecting a response.

Materials & experimental systems

n/a	Involved in the study
☒	☐ Antibodies
☒	☐ Eukaryotic cell lines
☒	☐ Palaeontology and archaeology
☐	☒ Animals and other organisms
☒	☐ Clinical data
☒	☐ Dual use research of concern
☒	☐ Plants

Methods

n/a	Involved in the study
☒	☐ ChIP-seq
☒	☐ Flow cytometry
☒	☐ MRI-based neuroimaging

Animals and other research organisms

Policy information about studies involving animals; ARRIVE guidelines recommended for reporting animal research, and Sex and Gender in Research

Laboratory animals	We used data from a previous study which made observations on bees in honeybee colonies in observation hives. For details see https//:doi.org/10.1111/1365-2664.14011
Wild animals	Study did not involve wild animals
Reporting on sex	All foraging honeybees (workers) are female
Field-collected samples	Study did not involve samples collected from the field
Ethics oversight	No ethics permission required. This paper uses data from a previous study, for details on data collection see https://doi.org/10.1111/1365-2664.14011

Note that full information on the approval of the study protocol must also be provided in the manuscript.

Plants

Seed stocks	NA
Novel plant genotypes	NA
Authentication	NA
